# Environmental Predictors of Seabird Wrecks in a Tropical Coastal Area

**DOI:** 10.1371/journal.pone.0168717

**Published:** 2016-12-16

**Authors:** Davi Castro Tavares, Jailson Fulgencio de Moura, Salvatore Siciliano

**Affiliations:** 1 Laboratório de Ciências Ambientais, Universidade Estadual do Norte Fluminense, Campos dos Goytacazes, RJ, Brasil; 2 Systems Ecology Group, Leibniz Center for Tropical Marine Ecology, Bremen, Germany; 3 Instituto Oswaldo Cruz, FIOCRUZ, Rio de Janeiro, RJ, Brasil; Hawaii Pacific University, UNITED STATES

## Abstract

Beached bird surveys have been widely used to monitor the impact of oil pollution in the oceans. However, separating the combined effects of oil pollution, environmental variables and methodological aspects of beach monitoring on seabird stranding patterns is a challenging task. The effects of a comprehensive set of oceanographic and climatic variables and oil pollution on seabird strandings in a tropical area of Brazil were investigated herein, using two robust and innovative methods: Generalized Linear Mixed Models and Structural Equation Modeling. We assessed strandings of four resident seabird species along 480 km of beaches divided into 11 sampling areas, between November 2010 and September 2013. We found that increasing the distance from the nearest breeding island reduce the seabird stranding events. Storm activity and biological productivity were the most important factors affecting the stranding events of brown boobies *Sula leucogaster*, Cabot’s terns *Thalasseus acuflavidus* and kelp gulls *Larus dominicanus*. These species are also indirectly affected by warm tropical waters, which reduce chlorophyll-a concentrations. Beach surveys are, thus, useful to investigate the mortality rates of resident species near breeding sites, where individuals are more abundant and exposed to local factors associated with at-sea mortality. In contrast, conservation actions and monitoring programs for far-ranging seabird species are needed in more distant foraging areas. Furthermore, beach monitoring programs investigating the impact of oil pollution on seabirds need to account for the effects of environmental factors on stranding patterns. The present study also demonstrated that seabirds inhabiting tropical coastal waters are sensitive to climate conditions such as adverse weather, which are expected to increase in frequency and intensity in next decades.

## Introduction

Seabirds are among the most vulnerable groups of marine vertebrates, with 97 of the 346 living species globally threatened, and the populations of 162 species showing declines [1, 2]. These organisms are sensitive to habitat alterations and anthropogenic disturbances, and, for this reason, are used as indicators of environmental quality and health [3–5]. Seabirds die at sea and their corpses are subsequently deposited along beaches. Since it is impossible to monitor seabird deaths at sea, mortality is evaluated based on carcass deposition patterns along beaches [6, 7]. Carcasses can be easily sampled to investigate the processes associated with mortality. Beached bird surveys have been widely used to monitor the impact of oil pollution in the oceans since the beginning of the twentieth century [8–10]. This scientific approach is also useful to investigate a number of threats to seabirds across the world’s oceans [11], such as storms and adverse weather conditions [12], lack of food under decreased productivity conditions [7], bycatch [13, 14], entanglements and ingestion of debris [15, 16], diseases [17] and contamination by chemical pollutants [18, 19].

Unravelling the effects of oceanographic, climatic and anthropogenic factors on seabird mortality is crucial to understand the main factors that can lead different species to population declines [20]. However, separating the combined effects of environmental variables, oil pollution and methodological aspects of beach surveys on seabird mortality is a challenging task [21]. Oils spills can affect seabird survival, inducing hypothermia, starvation, drowning or dehydration [6]. In contrast, specific oceanographic and climatic conditions may have contrasting effects on different seabird species [11, 22]. Strong winds reduce the ability of diving seabirds to chase food [23], although at the same time, may favour movements among food patches by facilitating energy-efficient flying [24, 25]. Cold nutrient-rich waters that surface during upwelling processes may benefit seabirds by increasing prey availability [7, 26], but may also induce thermoregulatory stress [6, 27]. Overall, the effects of increased frequency and intensity of extreme climatic events on seabirds are complex and remain poorly understood, particularly in tropical areas [4, 28–31].

Here we use beached bird surveys to investigate the effects of oceanographic and climatic variables, oil pollution and methodological aspects of beach monitoring programs on seabird strandings off the Brazilian coast, in the tropical Atlantic Ocean. Our working hypothesis is that seabirds are mostly affected by prey availability, sea surface temperature, and storm intensity, since these variables may affect their body condition and survival abilities [7, 11, 27]. Our study is the first to examine seabird strandings as a function of a comprehensive number of environmental variables and oil pollution along the Brazilian coast.

## Materials and Methods

### Study site

**Fig 1:**
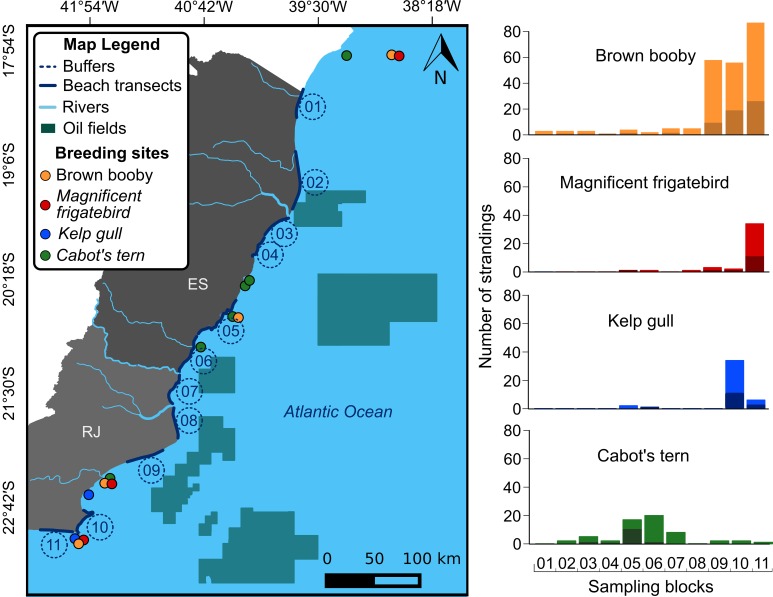
Panel summarizing the study design and seabird stranding events. The left panel shows the 11 transects covered to record seabird carcasses and adjacent buffers from where we extracted the average values of the variables used to predict stranding events along the South-eastern coast of Brazil. The right panel shows histograms indicating strandings for the four resident seabird studied species along the 11 transects. Dark and light shades indicate juveniles and adults, respectively.

Seabird carcasses were recovered during a systematic daily beach monitoring programme carried out along 11 sites within 480 kilometres of the Southeastern Brazilian coast (from 18°S to 23°S) (Fig 1). The study site is an ecologically important area that harbours threatened and rare marine species [32–35]. The Brazil Current prevails during the austral summer (December-March), characterized by warm (> 24°C) and nutrient-poor waters, while the Malvinas Current prevails during the austral winter (June-September), characterized by downwelling winds and stormy weather conditions [36]. An intense upwelling of the deep South Atlantic Central Water (SACW) affects the area between 21°S and 23°S from September (late winter) to April (autumn), when cold (<18°C) and nutrient-rich waters prevail [37]. In addition, the study area is influenced by intense oil and gas exploration and production activities, including the largest oil field in Brazil, known as the Campos Basin (Fig 1) [38].

### Ethics statement

This study comprised the collection of dead stranded seabirds under the protocol and regulations established by the National Center for Bird Conservation Research–CEMAVE (*Centro Nacional de Pesquisa para a Conservação das Aves Silvestres*), coordinated by the Brazilian Ministry of Environment–MMA (*Ministério do Meio Ambiente*) (http://www.icmbio.gov.br/cemave/). The study was approved by the Chico Mendes Institute for Biodiversity Conservation–ICMBio (*Instituto Chico Mendes de Conservação da Biodiversidade*), and conducted under SISBIO license #32550–2. The four seabird species studies are categorized as “Least Concern” on the IUCN Red List of Threatened Species (http://www.iucnredlist.org).

### Beach surveys

We have followed the sampling protocol recommended by previous studies assessing the efficiency of beached bird surveys in monitoring mortality sources [7, 11, 39, 40]. Seabird carcasses were collected during a daily beach monitoring program conducted between November 2010 and September 2013. Most of the beach monitoring programs worldwide are conducted monthly or weekly, but bird remains can be quickly removed from stranding locations on the beach due to tide variation, scavengers and beach cleaning activities [41, 42]. About 30% of the carcasses found washed ashore can be removed from beaches in a single day [43]. Thus, we conducted daily surveys to reduce the bias caused by carcass removal. In specific areas, a team of trained technicians conducted surveys along the high tide line along the 11 sampling transects, starting at dawn (Fig 1). The lengths of the transects remained fixed during the study period. From a total of 480 km travelled every day, 110.3 km of beaches were covered by vehicle, with similar performance compared to foot-based monitoring in detecting carcasses (S1 Fig). Tidal height in the study area does not show high variability, usually ranging from 0.5 m (low tide) to 0.8 m (high tide), not leading to substantial variations throughout the beach sampling areas. Carcasses were systematically collected or removed from beaches to avoid recounting. The analyses only included fresh carcasses, in order to reduce the bias caused by long drifting periods. We determined the stage of decomposition based on the scale available at Geraci and Lounsbury [44], where stage 1 represents live animals and stage 5 indicates mummified carcasses. We considered fresh all the intact specimens with no missing body parts or exposed skeleton (stage 1–4). In addition, records of specimens with evidence of mortality associated to bycatch (i.e. lines and hook) were excluded, based on the necropsy records, since the magnitude of fishing activities along the studied region was not always evaluated.

### Studied species

This study included seabird species that breed along the Brazilian coast since: i) migratory influxes may increase stochastic mortality and add bias in the assessment of stranding patterns [11]; and ii) migratory species may be affected by external factors to the study area [7]. The list of seabird species recorded during the study is displayed in S1 Table. We considered four seabird species: the brown booby *Sula leucogaster*, the magnificent frigatebird *Fregata magnificens*, the kelp gull *Larus dominicanus*, and Cabot’s tern *Thalasseus acuflavidus*. The brown booby, the magnificent frigatebird, and the kelp gull breed mainly on coastal islands located in the southern limit of the study area, while Cabot’s terns breed on islands in the north (Fig 1). The four species forage and are more abundant within 20 km from the breeding sites [45–48]. At the study site, *S*. *leucogaster*, *F*. *magnificens* and *L*. *dominicanus* breed throughout the entire year, with no marked reproductive peaks [48, 49]. *Thalasseus acuflavidus* aggregate to reproduce from April to October [46]. The stranding events of the studied seabird species throughout the study months and years are displayed in S2 Fig.

### Predictor variables

#### Satellite-derived measurements

To examine the most important factors influencing seabird strandings, we obtained satellite-derived measurements of a number of oceanographic and climatic variables from November 2010 to September 2013. Daily data were downloaded from NOAA Environmental Research Division's Data Access Protocol—ERDDAP (freely available at http://coastwatch.pfeg.noaa.gov/erddap). The included oceanographic variables were wave height and period from the Global Wave Model (WaveWatch III); water depth from the 1 arc-minute global relief model (ETOPO1); winds and currents zonal and meridional components from the microwave advanced scatterometer (ASCAT) on-board the satellite Metop-A (meteorological operational satellite programme); and sea surface temperature, chlorophyll-a concentrations and Diffuse Attenuation Coefficient at the 490 nm wavelength from the Moderate Resolution Imaging Spectroradiometer (MODIS). We also measured upwelling intensity using the upwelling index (UI) based on the Ekman's theory of mass transport of surface water caused by wind stress and the Coriolis force [50, 51]. We calculated the UI by means of UI = −(sin(θ−π/2)Q_x_+cos(θ−π/2)Q_y_), where θ is the angle between the landward side of the coastline and a vector pointing north, while Qx and Qy are the zonal and meridional components of Ekman’s transport [52]. Positive and negative values imply in upwelling and downwelling, respectively [53]. The environmental variables were extracted for circular buffers of 30 km in diameter, which reflect the habitats used by the examined seabird species (Fig 1) [5, 46, 48]. In addition, the recoveries of drifting corpses are markedly reduced when these objects are released at distances greater than 30 km from the shore of the studied region [54]. Buffers were positioned as close as possible to the edge of the continental shelf of the sampling areas. We calculated monthly averages for the predictor variables in each buffer [36]. After 35 months of surveys in 11 areas, we computed and analysed data from 385 sampling units.

#### Storm activity and adverse weather conditions

Storm events may increase seabird mortality due to direct trauma caused by waves and wind stress [55] leading to reduced foraging performance in rough sea conditions [12, 56]. We used wave height, wave period and river outflow as metrics for storm intensity and adverse weather conditions [11]. River outflow indicates rainfall periods, which may increase seabird mortality due to hypothermia [57]. We collected daily data on the river outflow from the hydro-meteorological stations under the responsibility of the Brazilian National Water Agency (ANA) (freely available at http://www.snirh.gov.br/hidroweb/). We also analysed the sea surface temperature, since this variable is an indicator of climatic variability and seabirds are sensitive to extreme temperatures, due to thermal stress [29, 58].

#### Prey availability

The decrease of prey availability may induce seabird mortality through starvation [7]. We assessed chlorophyll-a concentrations and the upwelling index because these variables indicate biological productivity and may serve as a metric for prey availability [7, 59, 60]. The Diffuse Attenuation Coefficient at the 490 nm wavelength served as a proxy for water turbidity, as this variable may affect prey detectability by seabirds [56].

#### Winds and currents

Bird corpses drift until they are deposited on shore [27]. Wind speed and direction seem to be the most important factors influencing carcass drifting [61, 62]. We calculated wind speed and direction from the zonal and meridional components using the conversion formulas available from Long [63]. We computed the onshore wind frequency by summing the proportion of onshore winds for each month based on daily-averaged estimates [64]. Onshore winds are defined as any wind parallel or toward the beach [27]. In addition, we included zonal and meridional current components in the analyses.

#### Oil spills

To investigate the effects of oil pollution on seabird strandings, we collected available data on the monthly volume of oil spills for each of the 11 circular buffers along the study area (S3 Fig). Oil spill information has been systematically recorded and notified by the Brazilian Institute for the Environment and Renewable Natural Resources–Ibama.

#### Distance from breeding islands

The probability of seabird stranding events may be affected by stochastic mortality according to variability of at-sea abundances [11]. Exposure probability models in which seabird strandings are assessed as a proportion of at-sea densities may provide more accurate estimations of at-sea mortality [6, 11]. However, to monitor at-sea densities is expensive and logistically challenging, making this unsuitable for developing countries such as Brazil. However, the seabirds species examined herein are more abundant and forage within 20 km from breeding sites, where they aggregate to reproduce and forage throughout the year [3, 5, 46, 56, 65, 66]. Thus, we used the linear distance between the nearest breeding island and the centre of surveyed transects as a metric for seabird at-sea abundance, i.e., the level of exposure of these organisms to local potential causes of death (i.e. oil pollution, storms, extreme temperatures). We collected data on breeding island for each of the bird studied species by reviewing specific literature (S2 Table). We measured the distance between each surveyed transect and the nearest breeding islands using a vector-based tool in QGIS 2.12.

#### Surveyed distance

The number of recovered carcasses is expected to be positively correlated with the surveyed distance during beach monitoring [40]. To investigate the effects of the surveyed distance on the numbers of recovered beached birds, 11 sampling transects were defined, with fixed distances (minimum = 13.8 km and maximum = 68.2 km).

### Statistical analyses

We organized and analysed data on seabird strandings and predictive predictor variables at a monthly scale in order to cover a feasible timespan in which seabird deaths at sea could be reflected in beaches due to carcass drift and delayed mortality [6], and to reduce excessive zeroes in data as a consequence of a low number of strandings. Therefore, 11 sampling sites were monitored during 35 months, totalling 385 observations.

We carefully explored the data in order to detect statistical issues such as outliers, collinearity, zero inflation, heterogeneity of variance and dependence of observations following the protocol provided by Zuur et al. [67]. We performed a Principal Component Analysis (PCA) on predictor variables in order to explore the covariance structure in the dataset. We assessed collinearity using the Variance Inflation Factor (VIF) and deleted each variable with a high VIF value until all remaining VIFs were below 3 [67]. Two variables were removed from the core statistical analysis, water depth and the Diffuse Attenuation Coefficient at the 490 nm wavelength, due to VIF values greater than 3 (S3 Table).

We examined the most important variables that affect the stranding events of the four studied seabird species using Generalized Linear Mixed Models (GLMMs) [68]. This technique allowed for the identification of non-normal response variables and spatial dependence of observations [69]. We fitted models to predict stranding events for each of the seabird species studied, with predictor variables set as fixed effects and sampling areas included as random effects, thus accounting for spatial correlations [3]. Since *T*. *acuflavidus* reproduces mainly between April and October [46], models for this species included interactions between season and distance from breeding islands. For each of the investigated species, we fitted models assuming binomial errors, considering the incidence of bird carcasses in a sampling transect as the response variable. The binomial errors were the most appropriate for the data, because stranding events were scarce. Nevertheless, the presence of one or few carcasses in a transect indicates a considerable number of deaths at-sea, since there is evidence that only 1–10% of floating corpses at sea are deposited on Brazilian shores and other regions worldwide [54, 62, 70]. Predictor variables were scaled and centered before being used in the modeling.

We fitted models using the Gauss-Hermite Quadrature to estimate parameters. For each species, we fitted at least 10 predictive models step-by-step, reducing from a full model including all predictor variables [68]. Model selection was based on Akaike’s information criterion–AIC_c_ corrected for small sample sizes [71]. Since small differences in AIC scores indicate very similar performances, we adopted a model averaging with shrinkage and set a cut-off of 2 AIC_c_, since estimates from models with higher AIC_c_ scores tend to be spurious [72]. We evaluated the predictive performance of the averaged models using the area under the Receiver Operating Characteristic (ROC) curves (AUC). The AUC values ranged from 0 to 1; model performance is considered good with AUC scores above 0.80, and excellent with AUC scores above 0.90 [73].

We also computed the importance of each variable influencing seabird strandings as the sum of the Akaike weights in a set of models randomly generated from the full model [71]. Models with larger weights better approximate the data [71]. Thus, we computed the importance by summing weights only at the 95% confidence set of models ranked from the largest to the smallest weights [74].

We performed Structural Equation Modeling (SEM) [75] in order to confirm the results obtained with the Generalized Linear Mixed Models. This method is suitable to examine causal relationships of direct and indirect effects of predictor on response variables [76]. In addition, SEM allows for the estimation of composite variables not directly measured in the study (also called latent variables), by including two or more observed variables [77]. For each species, we fitted models with significant predictor variables and with importance values greater than 0.70 according to the GLMMs and variables directed linked to the hypothesis of this study, namely, sea surface temperature, chlorophyll-a concentrations, wave height and river outflow. A composite term of distance from breeding islands and stranding events of both adults and juveniles were included as a response variable for each species, except for Cabot’s terns, for which adults and juveniles were merged to prevent trivial models due to the low incidence of juveniles. Model parameters were estimated using diagonally weighted least squares (DWLS), appropriate for binary response variables [78, 79]. All predictor variables were natural log-transformed. We evaluated model goodness-of-fit with a robust chi-square test [76]. Non-significant chi-square values (p > 0.05) indicate the model fits the data relatively well. We also used a multiple additional indicators of model fits, including: Comparative Fit Index–CFI (values > 0.95 indicate good model fits); root mean square error–RMSE (values < 0.06 indicate good model fits); and weighted root mean square residual–WRMR (values < 0.90 indicate good model fits) [80–83].

All the statistical analyses were carried out using the R software (version 3.0.2), using the packages ‘lme4’ for model fit, ‘bbmle’ for calculating AIC_c_ values, ‘pROC’ to evaluate model predictive performance and ‘MuMIn’ for model averaging and estimations of variable importance. We assessed collinearity between exploratory variables with the ‘corvif’ function, provided by Zuur et al. [69]. For the structural equation modeling, we used the packages ‘lavaan’ [84] and ‘semPlot’ [85].

## Results

From November 2010 to September 2013, a total of 192 stranding events were documented, including 99 brown boobies *S*. *leucogaster* (52%), 43 Cabot’s terns *T*. *acuflavidus* (22%), 28 kelp gulls *L*. *dominicanus* (15%), and 22 magnificent frigatebirds *F*. *magnificens* (11%). Two oiled birds (*L*. *dominicanus and S*. *leucogaster*) were documented from a total of 372 recovered carcasses, producing an oiling rate equal to 0.5% (i.e. oiled birds/total birds). The oiled specimens were found in February and November 2012, respectively. In February, a total of 7,112 L of oil were released into the sea, while in November a total of 1,520 L of oil was released. These oil spills were documented at about 100 km from the nearest breeding island (S3 Fig). The largest oil spill was documented in November 2011 at 120 km from transect ‘08’ (21°53'21.00" S, 39°49'41.00" W), releasing 477,000 L of oil in the sea (S3 Fig). Other episodes released relatively small volumes of oil (< 50,000 L).

**Fig 2:**
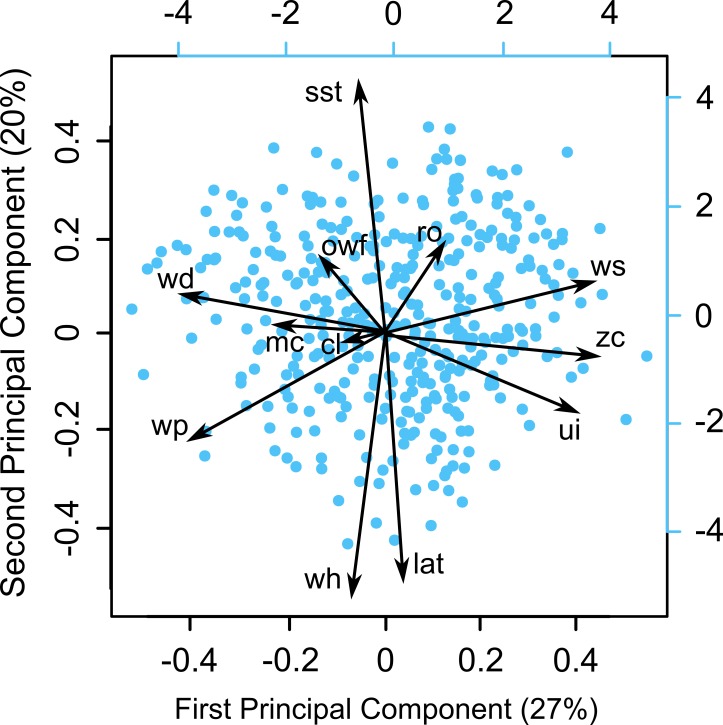
Principal Component Analysis demonstrating the relationship of the predictor variables. Dots indicate observations. Abbreviations indicate: chlorophyll-a concentrations (cl), latitude (lat), meridional currents (mc), onshore wind frequency (owf), river outflow (ro), sea surface temperature (sst), upwelling index (ui), wave height (wh), wave period (wp), wind direction (wd), wind speed (ws), and zonal currents (zc).

The first and the second principal components of the PCA explain, respectively, 27% and 20% of the total variance in the data (Fig 2). Increasing the latitude increased wave height and reduced sea surface temperature. Other variables showed no marked correlations.

### Generalized Linear Mixed Models

**Fig 3:**
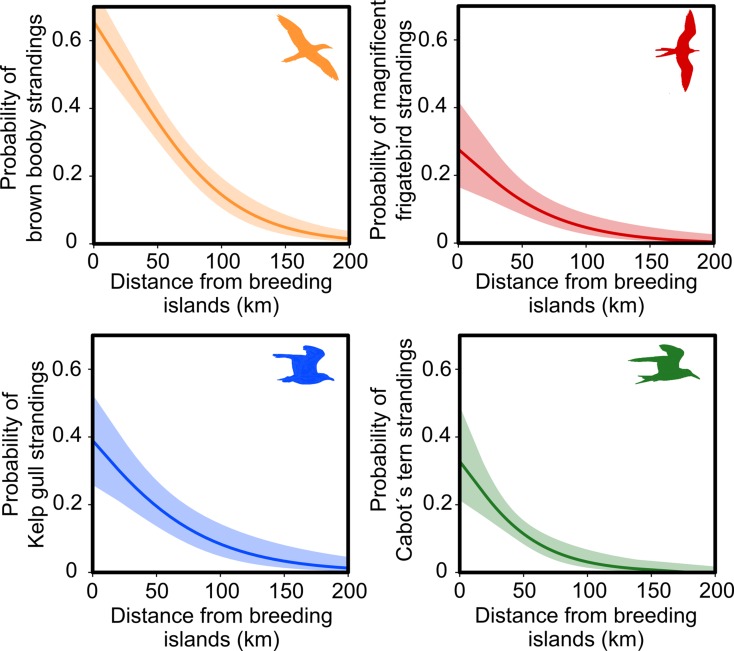
Responses of seabird strandings to the distance from the nearest breeding island in Brazil. Responses were obtained with Generalized Linear Models. Shaded areas indicate 95% confidence intervals.

The sets of models for predicting the stranding patterns of the investigated species differed in terms of complexity, although the distance from the nearest breeding island was included in all the best models (Table 1). In addition, the species showed specific responses to oceanographic and climatic variables, as well as to the volume of oil spills (Table 2). The probability of strandings of the four seabird species is considerably reduced in beaches located over 50 km from breeding islands (Fig 3). The responses of seabird strandings to significant environmental variables are displayed in S4 Fig.

**Table 1:** Ranking of the best model fits. The selection is based on the second-order Akaike’s information criterion (AIC_c_) corrected for small sample sizes (see Materials and Methods). The Generalized Linear Mixed Models were fitted with binomial errors for predicting seabird strandings as functions of environmental variables and oil pollution along the coast of Brazil.

Models	AIC_c_	ΔAIC_c_	w_*i*_
***Brown booby***			
wh + dbi + ds	311.7	0.0	0.23
wh + dbi	311.7	0.0	0.22
wh + wd + dbi + ds	312.1	0.4	0.18
sst + wh + wd + dbi + ds	313.3	1.6	0.10
wh + dbi + sst	313.4	1.7	0.09
***Magnificent frigatebird***			
owf + os + dbi	126.2	0.0	0.28
owf + ro + dbi	127.5	1.3	0.15
owf + ro + os +dbi + ds	127.5	1.3	0.14
owf + os + dbi + ds	128.1	1.9	0.11
***Kelp gull***			
sst + cl + dbi + ds	120.7	0.0	0.44
sst + cl + wp + dbi + ds	121.6	0.9	0.28
***Cabot's tern***			
sst + ro + wh + os + dbi * bp	227.6	0.0	0.18
sst + cl + ro + wh + os + dbi * bp	227.8	0.2	0.16
mc + sst + ro + wh + ws + os + dbi * bp	228.2	0.6	0.14
mc + sst + cl + ro + wh + ws + os + dbi * bp	228.7	1.1	0.11
sst + ro + wh + ws + os + dbi * bp	228.8	1.2	0.10
sst + cl + ro + wh + os + dbi * bp	229.4	1.7	0.08

Abbreviations indicate: chlorophyll-a concentrations (cl), distance from breeding islands (dbi), breeding period (bp), surveyed distance (ds), meridional currents (mc), oil spills (os), onshore wind frequency (owf), river outflow (ro), sea surface temperature (sst), wave height (wh), wave period (wp), wind direction (wd) and wind speed (ws). AIC_c_ = Second-order Akaike’s information criterion corrected for small sample sizes, ΔAIC_c_ = difference in AIC_c_ score between ranked models; w_*i*_ = AIC weights.

**Table 2:** Model-averaged parameter estimates. The Generalized Linear Mixed Models were fitted with binomial errors. Predictor variables are ordered according to importance scores (see Materials and Methods).

Predictor variables	β	95% CI lower	95% CI upper	P-value	IMP	AUC
***Brown booby***						
Wave height	0.49	0.01	0.98	0.04	0.82	0.87
Distance from breeding islands	-0.98	-1.68	-0.28	< 0.01	0.82	
Surveyed distance	-0.32	-1.19	0.15	0.39	0.47	
Wind direction	-0.09	-0.62	0.13	0.60	0.37	
Sea surface temperature	0.05	-0.31	0.70	0.76	0.32	
***Magnificent frigatebird***						
Distance from breeding islands	-1.42	-2.37	-0.46	< 0.01	0.99	0.89
Onshore wind frequency	-0.63	-1.06	-0.21	< 0.01	0.77	
Oil spills	-1.45	-5.20	1.50	0.39	0.77	
River outflow	-2.03	-16.16	6.68	0.65	0.64	
Surveyed distance	0.03	-1.03	1.20	0.93	0.27	
***Kelp gull***						
Distance from breeding islands	-5.53	-9.25	-1.81	< 0.01	1.00	0.94
Surveyed distance	2.78	0.66	4.90	0.01	0.92	
Chlorophyll-a	-1.36	-2.39	-0.31	0.01	0.90	
Sea surface temperature	-0.68	-1.31	-4.61	0.03	0.71	
Wave period	0.10	-0.23	0.76	0.61	0.4	
***Cabot's tern***						
Distance from breeding islands	-0.83	-1.35	-0.30	< 0.01	1.00	0.84
Surveyed distance	0.80	0.36	1.24	< 0.01	0.99	
River outflow	0.59	0.21	0.97	< 0.01	0.96	
Non-breeding period	-1.39	-2.67	-0.10	0.03	0.91	
Oil spills	0.34	0.09	0.60	< 0.01	0.75	
Wave height	-0.49	-0.93	-0.05	0.03	0.63	
Chlorophyll-a	0.14	-0.15	0.75	0.52	0.56	
V current	-0.13	-0.92	0.08	0.58	0.55	
Sea surface temperature	-0.36	-2.84	-1.64	0.18	0.40	
Wind speed	-0.17	-0.84	0.20	0.49	0.40	
Non-breeding period * dbi	-0.10	-1.20	1.00	0.86	-	

β = parameter estimates for slopes (coefficients); CI = Confidence interval; IMP = variable importance (the sum of the Akaike weights for each variable in a set of models randomly generated from the full model); AUC = area under ROC curve; dbi = distance from breeding islands.

#### Brown booby

The averaged model included five predictor variables for brown booby strandings, of which distance from breeding islands and wave height were significant (P < 0.05). The wave height and distance from breeding islands showed importance values of 0.85 and 0.82, respectively (Table 2). The slopes indicate that stranding events of brown boobies are positively correlated with wave height (Table 2). The averaged model showed good predictive performance (AUC = 0.87).

#### Magnificent frigatebird

The averaged model included five variables to predict magnificent frigatebird strandings, of which distance from breeding islands and the frequency of onshore winds were significant (P < 0.05). The slopes indicate that stranding events of magnificent frigatebirds are negatively correlated with distance from breeding islands and the frequency of onshore winds (Table 2). The averaged model showed good predictive performance (AUC = 0.89).

#### Kelp gull

For kelp gulls, the averaged model included five variables, of which distance from breeding islands, surveyed distance, chlorophyll-a concentrations and sea surface temperature were significant (P < 0.05). The slopes indicate that stranding events of kelp gulls are positively correlated with the surveyed distance and negatively correlated with distance from breeding islands, chlorophyll-a concentrations and sea surface temperature (Table 2). The averaged model showed excellent predictive performance (AUC = 0.94).

#### Cabot’s tern

The averaged model to predict Cabot’s terns strandings included 10 variables, of which distance from breeding islands, surveyed distance, river outflow, volume of oil spills, sea surface temperature, wave height and wind speed were significant (P < 0.05). Cabot’s terns strandings were negatively correlated with distance from breeding islands, sea surface temperature, wind speed and wave height, and positively correlated with river outflow and chlorophyll-a concentrations (Table 2). The averaged model showed good predictive performance (AUC = 0.82).

### Structural Equation Models

**Fig 4:**
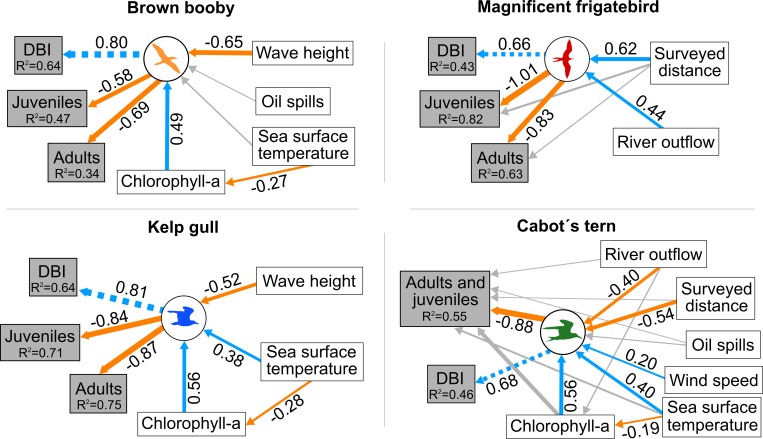
Best-fitting Structural Equation Models (SEMs) for stranding seabird wrecks in Brazil. Predictor variables are shown in white boxes, while the composite terms are shown in the circles. Response variables included in the composite term are displayed in grey boxes. Significant paths (P < 0.05) are presented in blue (positive effects) and in orange (negative effects). Non-significant paths (P > 0.05) are shown in grey. Numbers adjacent to arrows indicate path coefficient estimates. The larger the coefficient, the greater the magnitude of the relationship between the variables. The width of the arrows is proportional to the value of the standardized coefficients (comparable to each other). The model fit the data (S4 Table). The variance explained by the model (R^2^) is shown next to each response variable.

The structural equation modelling (SEM) allowed to test for direct and indirect effects of environmental variables on stranding events of the four species studied, based on the hypothesis of this study. The measures of goodness of fit indicate the models fit the data (S4 Table). In general, results from SEMs were consistent with those inferred by GLMMS. The best-fitting SEMs confirmed a negative relationship between the probability of strandings and the distance from breeding islands for both adults and juveniles of the investigated species (Fig 4).

#### Brown booby

The best-fitting SEM confirmed the positive effect of wave height in strandings events of this species (Fig 4). In addition, the SEM also revealed an indirect influence of sea surface temperature on strandings of both adults and juveniles. Specifically, increasing sea surface temperature reduces chlorophyll-a concentrations, in turn increasing the probability of stranding events.

#### Magnificent frigatebird

The best-fitting SEM revealed a negative effect of river outflow on stranding events of both adults and juveniles (Fig 4). In addition, surveyed distance was negatively correlated with strandings events of this species.

#### Kelp gull

Wave height showed a positive effect on stranding events of both adults and juveniles (Fig 4). In addition, the SEM revealed an indirect influence of sea surface temperature on strandings of both adults and juveniles. Increasing sea surface temperature reduced chlorophyll-a concentrations and increased the probability of kelp gull stranding events.

#### Cabot’s tern

The SEM confirmed the positive effect of river outflow and surveyed distance on stranding events for this species (Fig 4). Sea surface temperature directly and indirectly affected stranding events by reducing chlorophyll-a concentrations and increasing the probability of kelp gull stranding events. Increasing wind speed reduced the probability of stranding events.

## Discussion

Beach surveys are useful to investigate seabird mortality near breeding sites, where birds are abundant and exposed to local sources of mortality. The present study demonstrated that three out four of the studied species are sensitive to extreme climate conditions such as adverse weather (i.e. tropical storms), which are expected to increase in frequency and intensity in the next decades [86, 87]. Also, we demonstrate that the combination of environmental conditions, such as increased sea surface temperature, reduced biological productivity and adverse weather, is affecting seabird mortality at tropical sites. Overlooking the influence of these variables on stranding patterns may affect the reliability of beach monitoring to track oil pollution in the marine environment and detect trends in mortality events. Beach monitoring programs, thus, need to account for the environmental variables that affect bird mortality and stranding patterns, since this will increase the ability to detect trends in mortality factors.

The results indicate that the frequency of seabird strandings is higher in areas near breeding sites, where these organisms are more abundant. Increases in carcass deposition may be affected by stochastic mortality due to variations in at-sea abundances [11]. Thus, beach monitoring programs need to take into account metrics for variations in seabird at-sea densities. The findings presented herein are novel in revealing that information on seabird breeding sites may serve as a relatively rapid and inexpensive method to estimate stranding probabilities along beaches. In addition, the effect size of distance to the nearest breeding island varied across species, which may be explained, in part, by differences in foraging range. Kelp gull strandings showed abrupt declines with increasing distance from colonies (β = -5.53), reflecting the species’ foraging range restricted to inshore waters [56]. Thus, carcasses of these species are deposited in beaches close to breeding sites in the southern of the study area. The probabilities of brown boobies and Cabot’s terns stranding were affected to a lesser extent (β = -0.98 and β = -0.84, respectively). These species forage within 20–25 km from breeding sites [45, 46], and, therefore, their carcasses may be found in relatively distant beaches from their colonies. Quite puzzlingly, the effect size of distance from breeding islands for the magnificent frigatebird was pronounced (β = -1.42), but the species forage within 300 km from colonies [88]. Beach monitoring programs and conservation actions to protect seabirds may be focused on environments located within 50 km of breeding sites, where stranding incidence is higher than in remote areas. In contrast, conservation actions and monitoring programs for far-ranging seabird species are needed in distant foraging areas.

We documented an increased probability of stranding events of brown boobies, kelp gulls and Cabot’s terns under increased storm activity (i.e. wave height and river outflow). Seabirds show decreased ability to obtain food and spend more energy to survive in rough seas during stormy conditions [7, 11]. Reduced foraging success may lead these organisms to starvation and may, subsequently, increase the risk of mortality [89]. As a consequence of climate change, tropical storms are becoming more frequent and intense [87, 90, 91]. These events may increase seabird mortality in the future, with negative consequences for species with declining populations. The impact of tropical storms is expected to be more severe for migratory bird species, since they face energetic constraints during their long distance movements [92, 93]. For example, the first documented hurricane in Brazil occurred in 2007 and resulted in a mass mortality of Atlantic petrels *Pterodroma incerta*, with at least 354 individuals found up to 420 km inland and 1,100 m above the sea level [94]. High mortalities of the Manx’s shearwater *Puffinus puffinus* usually occur in Southeastern Brazilian coast during adverse weather [95]. In contrast, stormy conditions were not the main force influencing seabird strandings along the coast of California, where decreased productivity seems to be the main cause of wrecks [7].

The Structural Equation Models (SEMs) revealed that combined conditions of warm waters and reduced chlorophyll-a concentrations contribute to an increase in brown boobies, kelp gulls and Cabot’s terns stranding events. This finding indicates that bottom-up food web mechanisms that lurk beneath in Brazilian coast affect seabird wrecks. Parrish et al. [7] found that a strong effect of bottom-up food web processes in seabird beaching along the coast of California (EUA) is associated with changes in the timing and intensity of the upwelling. In contrast, we found no significant effect of upwelling intensity on chlorophyll-a concentrations throughout the study site. The bottom-up mechanism along Brazilian coast is more likely driven by the predominant tropical warm waters. In contrast, anomalous cold waters can reduce fish larvae survival, leading to decreased availability of seabird prey [96]. In Brazil, an extreme mortality event of southern migrant Magellanic penguins *Spheniscus magellanicus* occurred in 2008, when sea surface temperature negatively impacted fish larvae recruitment, subsequently reducing prey availability in the South Atlantic Ocean [97]. Newton et al. [11] demonstrated that the effect of decreased prey availability is stronger for migratory species (i.e. sooty shearwater *Ardenna grisea*), which deal with energetic constraints, when compared to resident species. Thus, the stress caused by climatic variability along the Brazilian coast may be a source of seabird wrecks.

Cabot’s terns stranding probability was affected by a number of factors, including sea surface temperature, river outflow and biological productivity (i.e. chlorophyll-a concentrations). This species is likely to be more sensitive to climate variability and human pollution. Cabot’s terns have smaller body size and shorter life spans than the other investigated species [56, 98]. Body size is positively correlated with longevity, since small birds need to feed more frequently than large birds to fulfil their high metabolic rates [99, 100]. Furthermore, thermoregulation capacity is correlated with body size, making small-bodied species more vulnerable to low temperatures and the negative effect of oiling in the heat insulating properties of feathers [6, 101]. The stranding events of Cabot’s terns were positively affected by river outflow. This variable is expect to affect foraging visibility by reducing water clarity [102, 103], although this seems not to be an ubiquitous pattern [104]. Cabot’s terns feed via surface plunging with limited access to fish below the water column [103] and, for this reason, exhibit narrower diet. Thus the species is more sensitive to declines in prey availability caused by increased water turbidity and adverse weather conditions, which disrupt surface foraging visibility [105].

The volume of oil spills was positively correlated only with Cabot’s terns stranding events. Quite puzzlingly, no oiled carcasses of this species were recovered. However, visible oiling may be not an accurate indicator of bird death due to this form of pollution, since the impacted individuals do not necessarily exhibit oiled feathers [106]. Birds may ingest oil by preening feathers, directly from the sea surface or accumulated in their food [107]. Oil ingestion impairs nutrient uptake and reduces the efficacy of the immune system, thereby reducing seabird survival ability [108]. The impact of oil spills on seabird mortality may, thus, be underestimated in beach monitoring programs when only examining oiled feathers. A better way to estimate the number of birds impacted by oil may be to analyse necropsied individuals, in which oil ingestion and oiled feathers can be assessed. At the study site, most episodes released relatively small volumes of oil, at least at 100 km from the breeding islands, which explain the low oiling rates of the studied coastal species. Neverthless, oil pollution is a problem in Brazil for other coastal species, such as the Magellanic penguin, which spends more time in the water and may serve as a better indicator of this form of sea pollution[109]. Curiously, the incidence of magnificent frigatebirds was negatively correlated with the volume of oil spills. Carcasses of this species were recorded mainly in southern area of the study site, where oil spills were less frequent and comprised of small volumes (S3 Fig), thus producing a significant statistical relationship with no biological meaning.

Most of the literature indicates that onshore winds positively affect the number of carcasses found washed ashore [61, 62, 110, 111]. Surprisingly, results herein demonstrate that onshore wind frequency was negatively correlated with the stranding events of magnificent frigatebirds. Similar results were documented by Wilhelm et al. [27]. The trajectory of drifting corpses may be influenced by other factors such as buoyance as a function of water temperature and salinity, and a force resulting from the combined effects of winds and currents [112, 113]. In the region studied, onshore winds prevail during winter months, when adverse sea conditions might increase the sinking probability of seabird carcasses [27]. Further investigations regarding the effects of winds on seabird stranding patterns are needed. In addition, the SEMs revealed a negative effect of river outflow on the strandings events of magnificent frigatebird. River discharges forces might surpass the effect of onshore wind direction on drifting carcasses by pushing them offshore [36].

## Conclusions

In summary, we demonstrated that increasing the distance from breeding islands considerably reduces the probability of strandings for the investigated seabird species. Areas located within 50 km of a breeding site should be primarily monitored to maximize carcass records of species with such relatively small foraging ranges. In contrast, conservation actions and monitoring programs for far-ranging seabird species are needed in distant foraging areas. Furthermore, not much attention has been dedicated so far to the combined effects that environmental and anthropogenic variables may have on seabird stranding patterns, particularly in tropical areas. We showed stranding events of resident seabirds are influenced by storm activity, and indirectly by warm tropical Brazilian waters, which reduce chlorophyll-a concentrations and prey availability. Storm activity in tropical areas is expected to increase in frequency and intensity in next decades as result of human-induced climate change. Thus, extreme environmental conditions may increase seabird mortality in the future.

## Supporting Information

S1 Fig Descriptive statistics of beached birds recovered during daily beach surveys conducted by foot and by vehicle. Number of carcasses recovered during surveys by foot (mean = 1.1, coefficient of variation = 178) and vehicle (mean = 0.73, coefficient of variation = 223). Total daily distance travelled by foot = 372 km and vehicle = 110.3 km.
(DOCX)

S2 Fig Temporal variation of stranding events of the four studied seabird species. Filled cells indicated the presence of carcasses along the study site, while empty cells indicate absence. Letters in black below each panel indicate breeding months, while letters in grey indicate non-breeding months.
(DOCX)

S3 Fig Episodes of oil spills and oiled carcasses recovered along the Brazilian coast (17° – 23° S), between November 2010 and September 2013. (DOCX)

S4 Fig Model fits. Predicted probabilities and 95% confidence intervals (shaded areas) of seabird strandings in response to significant environmental variables. The responses were obtained with Generalized Linear Mixed Models (GLMMs) fitted with binomial errors.
(DOCX)

S1 Table List of seabird species recorded during daily beached bird monitoring along Brazilian coast (17° – 23° S). (DOCX)

S2 Table Breeding sites of the four studied seabird species along the Brazilian coast (17°S-23°S). Black dots indicate the presence of species at the breeding sites.
(DOCX)

S3 Table Variance Inflation Factors for the predictor variables. (DOCX)

S4 Table The measures of goodness of fit of the structural equation models for seabird stranding events in Brazil. Model goodness of fit to the data was evaluated using multiple indicators: chi-square test (p-values > 0.05 indicate relatively good model fits); Comparative Fit Index (CIF, values > 0.95 indicate good model fits); root mean square error (RMSE, values < 0.06 indicate good model fits) and the weighted root mean square residual (WRMR, values < 0.90 indicate good model fits). The fit indices indicate the Structural Equation Models for all the species met the standard criteria: CFI > 0.96, RMSE < 0.05, and WRMR < 0.65.]
(DOCX)
