# Supplementary material for: Environmental Predictors of Seabird Wrecks in a Tropical Coastal Area
Source: PLoS One. 2016 Dec 16;11(12):e0168717. doi: 10.1371/journal.pone.0168717 (PMC5161483; doi:10.1371/journal.pone.0168717)
Supplement: S1 Fig — Number of carcasses recovered during surveys by foot (mean = 1.1, coefficient of variation = 178) and vehicle (mean = 0.73, coefficient of variation = 223). Total daily distance travelled by foot = 372 km and vehicle = 110.3 km. (DOCX) [file pone.0168717.s001.docx]

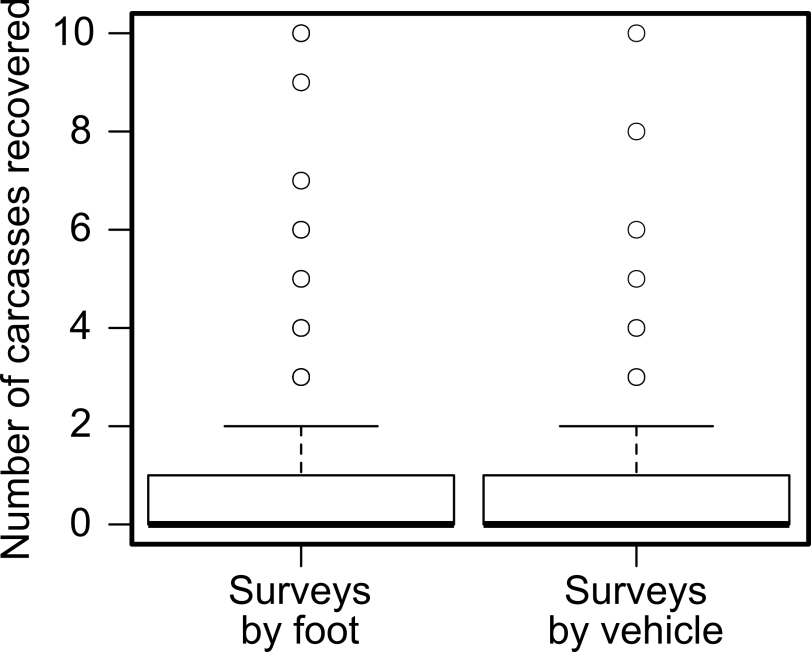


**S1 Fig. Descriptive statistics of beached birds recovered during daily beach surveys conducted by foot and by vehicle.** Number of carcasses recovered during surveys by foot (mean = 1.1, coefficient of variation = 178) and vehicle (mean = 0.73, coefficient of variation = 223). Total daily distance travelled by foot = 372 km and vehicle = 110.3 km.
