# Supplementary material for: Environmental Predictors of Seabird Wrecks in a Tropical Coastal Area
Source: PLoS One. 2016 Dec 16;11(12):e0168717. doi: 10.1371/journal.pone.0168717 (PMC5161483; doi:10.1371/journal.pone.0168717)
Supplement: S2 Fig — Filled cells indicated the presence of carcasses along the study site, while empty cells indicate absence. Letters in black below each panel indicate breeding months, while letters in grey indicate non-breeding months. (DOCX) [file pone.0168717.s002.docx]

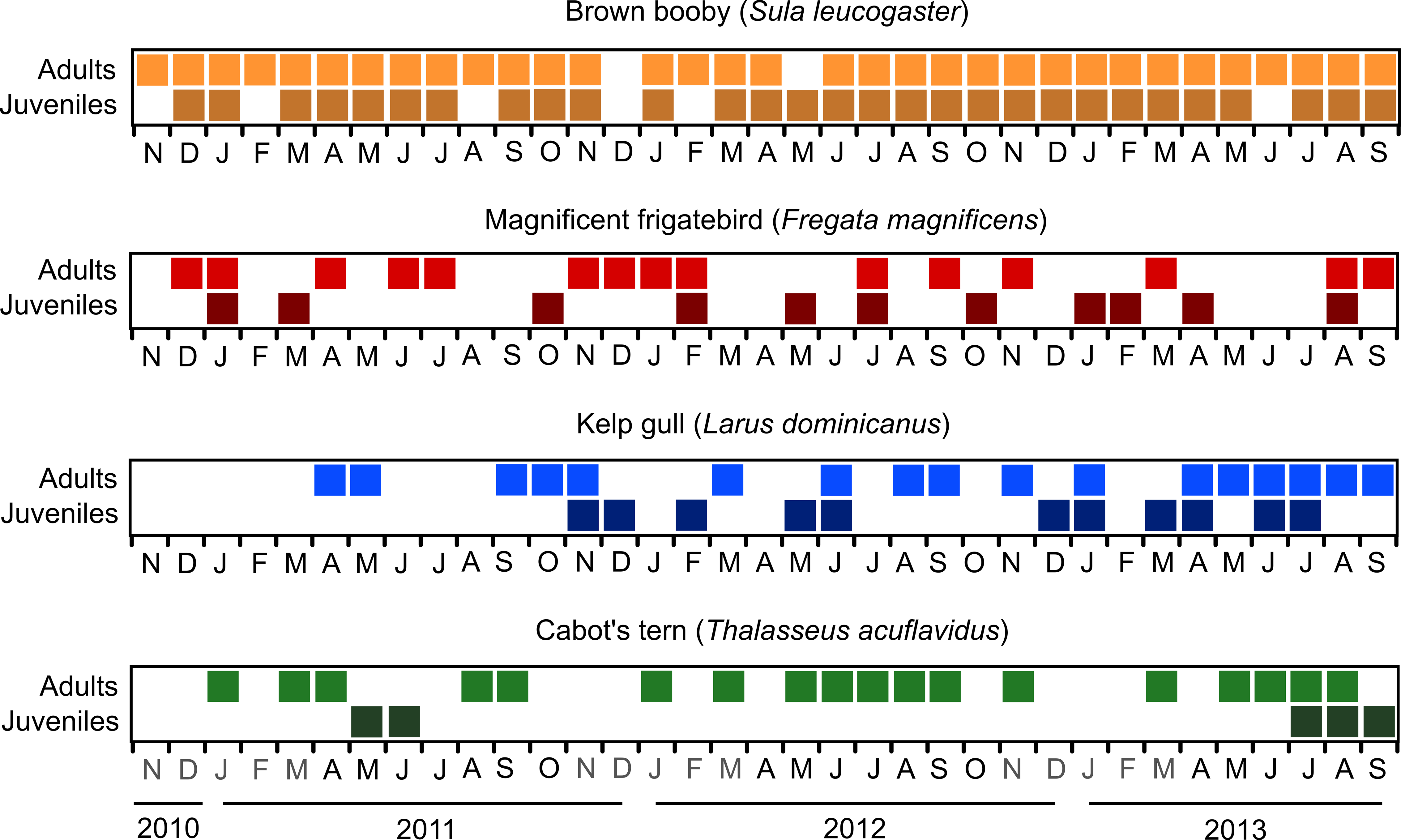


**S2 Fig. Temporal variation of stranding events of the four studied seabird species.** Filled cells indicated the presence of carcasses along the study site, while empty cells indicate absence. Letters in black below each panel indicate breeding months, while letters in grey indicate non-breeding months.
