# Supplementary material for: Environmental Predictors of Seabird Wrecks in a Tropical Coastal Area
Source: PLoS One. 2016 Dec 16;11(12):e0168717. doi: 10.1371/journal.pone.0168717 (PMC5161483; doi:10.1371/journal.pone.0168717)
Supplement: S3 Fig — (DOCX) [file pone.0168717.s003.docx]

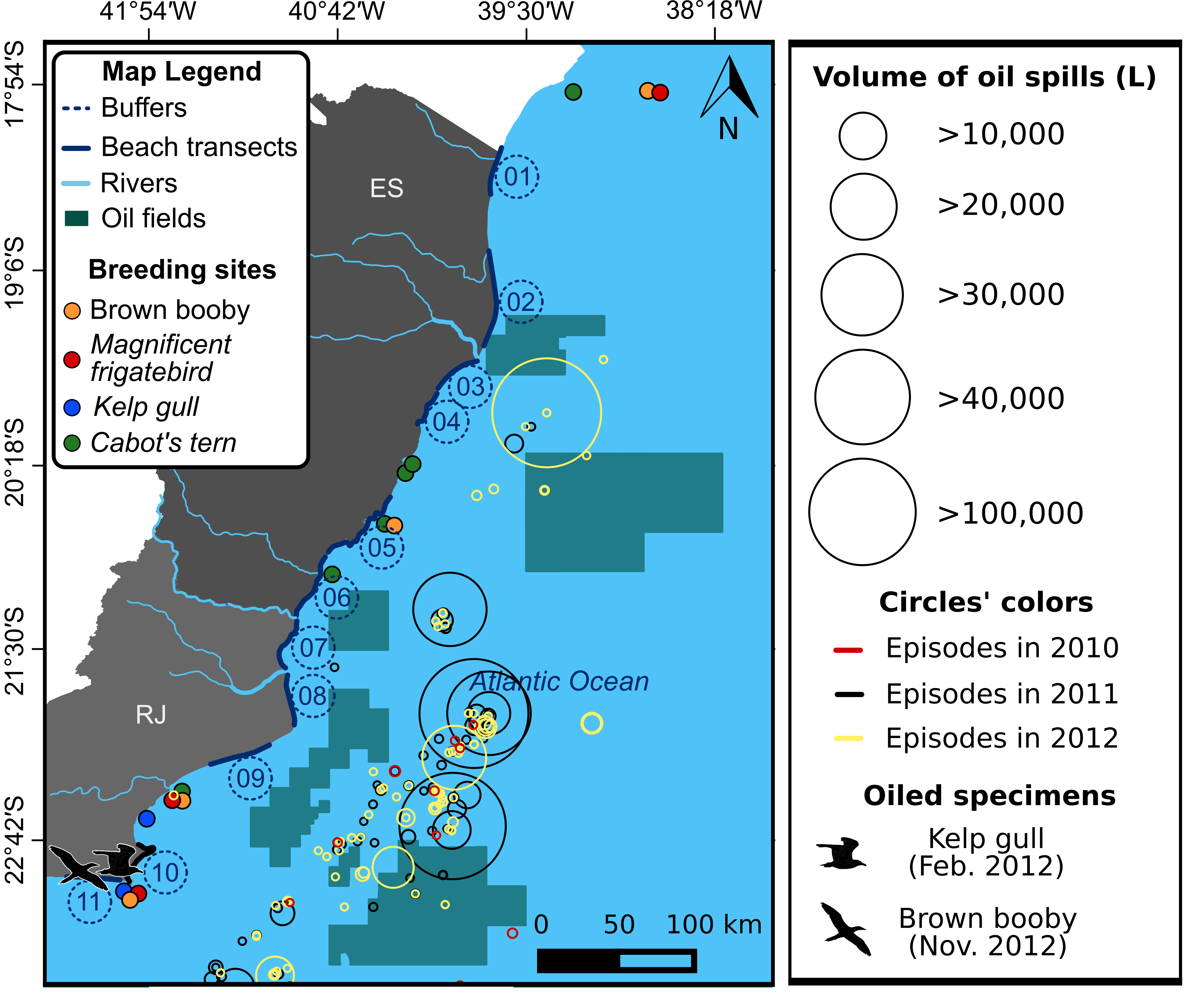


**S3 Fig. Episodes of oil spills and oiled carcasses recovered along the Brazilian coast (17° – 23° S), between November 2010 and September 2013**.
