# Supplementary material for: Environmental Predictors of Seabird Wrecks in a Tropical Coastal Area
Source: PLoS One. 2016 Dec 16;11(12):e0168717. doi: 10.1371/journal.pone.0168717 (PMC5161483; doi:10.1371/journal.pone.0168717)

**S4 Fig. Model fits.** Predicted probabilities and 95% confidence intervals (shaded areas) of seabird strandings in response to significant environmental variables. The responses were obtained with Generalized Linear Mixed Models (GLMMs) fitted with binomial errors.


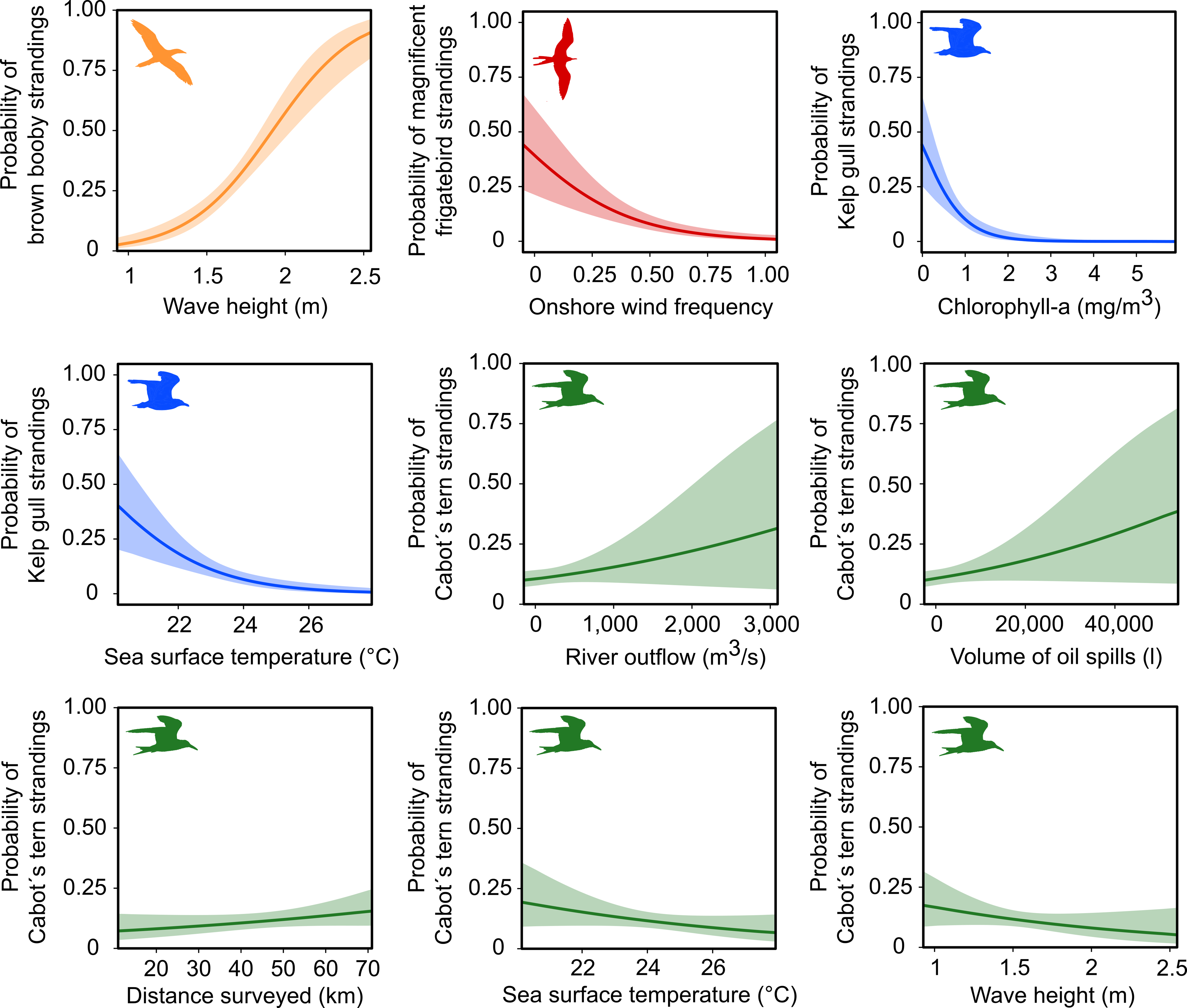

Supplement: S4 Fig — Predicted probabilities and 95% confidence intervals (shaded areas) of seabird strandings in response to significant environmental variables. The responses were obtained with Generalized Linear Mixed Models (GLMMs) fitted with binomial errors. (DOCX) [file pone.0168717.s004.docx]
