# Supplementary material for: Environmental Predictors of Seabird Wrecks in a Tropical Coastal Area
Source: PLoS One. 2016 Dec 16;11(12):e0168717. doi: 10.1371/journal.pone.0168717 (PMC5161483; doi:10.1371/journal.pone.0168717)
Supplement: S1 Table — (DOCX) [file pone.0168717.s005.docx]

**S1 Table. List of seabird species recorded during daily beached bird monitoring along Brazilian coast (17° – 23° S).** The species are ordered following [Piacentini et al. [1]](#_ENREF_1).

| **Taxa** | **English Name** | **Status** | **IUCN [**[**2**](#_ENREF_2)**]** | **Population trend [**[**2**](#_ENREF_2)**]** |
| --- | --- | --- | --- | --- |
| Sphenisciformes Sharpe, 1891 |  |  |  |  |
| Spheniscidae Bonaparte, 1831 |  |  |  |  |
| *Spheniscus magellanicus* (Forster, 1781) | Magellanic Penguin | Southern migrant | Near threatened | Declining |
| Procellariiformes Fürbringer, 1888 |  |  |  |  |
| Diomedeidae Gray, 1840 |  |  |  |  |
| *Phoebetria palpebrata* (Forster, 1785) | Light-mantled Albatross | Southern migrant | Near threatened | Declining |
| *Thalassarche chlororhynchos* (Gmelin, 1789) | Yellow-nosed Albatross | Southern migrant | Endangered | Declining |
| *Thalassarche melanophris* (Temminck, 1828) | Black-browed Albatross | Southern migrant | Near threatened | Declining |
| Procellariidae Leach, 1820 |  |  |  |  |
| *Fulmarus glacialoides* (Smith, 1840) | Southern Fulmar | Southern migrant | Least concern | Stable |
| *Daption capense* (Linnaeus, 1758) | Cape Petrel | Southern migrant | Least concern | Stable |
| *Pterodroma mollis* (Gould, 1844) | Soft-plumaged Petrel | Southern migrant | Least concern | Stable |
| *Pterodroma arminjoniana* (Giglioli & Salvadori, 1869) | Trindade Petrel | Resident | Vulnerable | Stable |
| *Procellaria aequinoctialis* Linnaeus, 1758 | White-chinned Petrel | Southern migrant | Vulnerable | Declining |
| *Procellaria conspicillata* Gould, 1844 | Spectacled Petrel | Southern migrant | Vulnerable | increasing |
| *Calonectris borealis* (Cory, 1881) | Cory's Shearwater | Northern migrant | Least concern | Declining |
| *Ardenna grisea* (Gmelin, 1789) | Sooty Shearwater | Southern migrant | Near threatened | Declining |
| *Ardenna gravis* (O'Reilly, 1818) | Great Shearwater | Southern migrant | Least concern | Stable |
| *Puffinus puffinus* (Brünnich, 1764) | Manx Shearwater | Northern migrant | Least concern | Declining |
| Suliformes Sharpe, 1891 |  |  |  |  |
| Fregatidae Degland & Gerbe, 1867 |  |  |  |  |
| *Fregata magnificens* Mathews, 1914 | Magnificent Frigatebird | Resident | Least concern | Increasing |
| Sulidae Reichenbach, 1849 |  |  |  |  |
| *Sula dactylatra* Lesson, 1831 | Masked Booby | Resident | Least concern | Declining |
| *Sula leucogaster* (Boddaert, 1783) | Brown Booby | Resident | Least concern | Declining |
| Charadriiformes Huxley, 1867 |  |  |  |  |
| Stercorariidae Gray, 1870 |  |  |  |  |
| *Stercorarius skua* (Brünnich, 1764) | Great Skua | Northern migrant | Least concern | Stable |
| *Stercorarius chilensis* Bonaparte, 1857 | Chilean Skua | Southern migrant | Least concern | Stable |
| *Stercorarius maccormicki* Saunders, 1893 | South Polar Skua | Southern migrant | Least concern | Stable |
| *Stercorarius pomarinus* (Temminck, 1815) | Pomarine Jaeger | Northern migrant | Least concern | Stable |
| *Stercorarius parasiticus* (Linnaeus, 1758) | Parasitic Jaeger | Northern migrant | Least concern | Stable |
| Laridae Rafinesque, 1815 |  |  |  |  |
| *Chroicocephalus cirrocephalus* (Vieillot, 1818) | Gray-hooded Gull | Resident | Least concern | Stable |
| *Larus dominicanus* Lichtenstein, 1823 | Kelp Gull | Resident | Least concern | Increasing |
| Sternidae Vigors, 1825 |  |  |  |  |
| *Anous stolidus* (Linnaeus, 1758) | Brown Noddy | Resident | Least concern | Stable |
| *Onychoprion fuscatus* (Linnaeus, 1766) | Sooty Tern | Resident | Least concern | Declining |
| *Sternula superciliaris* (Vieillot, 1819) | Yellow-billed Tern | Resident | Least concern | Stable |
| *Phaetusa simplex* (Gmelin, 1789) | Large-billed Tern | Resident | Least concern | Stable |
| *Sterna hirundo* Linnaeus, 1758 | Common Tern | Northern migrant | Least concern | Declining |
| *Sterna dougallii* Montagu, 1813 | Roseate Tern | Northern migrant | Least concern | Declining |
| *Sterna hirundinacea* Lesson, 1831 | South American Tern | Resident | Least concern | Declining |
| *Thalasseus acuflavidus* (Cabot, 1847) | Cabot's Tern | Resident | Least concern | Stable |
| *Thalasseus maximus* (Boddaert, 1783) | Royal Tern | Resident | Least concern | Stable |

**S1 Table References**

**1.** Piacentini VQ, Aleixo A, Agne CE, Maurício GN, Pacheco JF, Bravo GA, et al. Annotated checklist of the birds of Brazil by the Brazilian Ornithological Records Commitee. Revista Brasileira de Ornitologia. 2015;23(2):91-298.

**2.** BirdLife International. IUCN Red List for birds 2016. Available: <http://www.birdlife.org>.
