# Supplementary material for: Environmental Predictors of Seabird Wrecks in a Tropical Coastal Area
Source: PLoS One. 2016 Dec 16;11(12):e0168717. doi: 10.1371/journal.pone.0168717 (PMC5161483; doi:10.1371/journal.pone.0168717)
Supplement: S2 Table — Black dots indicate the presence of species at the breeding sites. (DOCX) [file pone.0168717.s006.docx]

**S2 Table. Breeding sites of the four studied seabird species along the Brazilian coast (17°S-23°S)**. Black dots indicate the presence of species at the breeding sites.

| **Breeding sites** | **State** | **Lat.** | **Long.** | **Sl** | **Fm** | **Ld** | **Ta** |
| --- | --- | --- | --- | --- | --- | --- | --- |
| **Arquipélago de Abrolhos** | Bahia | -17.96 | -38.7 | *●* | *●* |  |  |
| **Ilha da Coroa Vermelha** | Bahia | -17.96 | -39.2 |  |  |  | *●* |
| **Ilha dos Pacotes** | Espírito Santo | -20.35 | -40.25 |  |  |  | *●* |
| **Ilha de Itatiaia** | Espírito Santo | -20.36 | -40.27 |  |  |  | *●* |
| **Ilha Escalvada** | Espírito Santo | -20.7 | -40.4 | *●* |  |  | *●* |
| **Ilha Branca** | Espírito Santo | -21.00 | -40.78 |  |  |  | *●* |
| **Arquipélago de Santana** | Rio de Janeiro | -22.41 | -41.72 | *●* | *●* |  | *●* |
| **Arquipélago dos Trinta-réis** | Rio de Janeiro | -22.56 | -41.95 |  |  | *●* |  |
| **Ilha Comprida** | Rio de Janeiro | -22.86 | -41.94 | *●* |  | *●* |  |
| **Ilha dos Franceses** | Rio de Janeiro | -22.98 | -42.03 | *●* | *●* | *●* |  |
| **Ilha de Cabo Frio** | Rio de Janeiro | -22.99 | -41.99 | *●* | *●* | *●* |  |

Sl = *Sula leucogaster*, Fm = *Fregata magnificens*, Ld = *Larus dominicanus*, and Ta = *Thalasseus acuflavidus*. Data collected in the following references: [Vooren and Brusque [1]](#_ENREF_1), [Alves et al. [2]](#_ENREF_2), [Efe [3]](#_ENREF_3), [Efe et al. [4]](#_ENREF_4), and [Efe and Bonatto [5]](#_ENREF_5).

**S2 Table References**

1. Vooren CM, Brusque LF. As aves do ambiente costeiro do Brasil: biodiversidade e conservação. Rio Grande do Sul: Fundação Universidade Federal de Rio Grande; 1999. 58 p.

2. Alves VS, Soares ABA, Couto GS. Aves marinhas e aquáticas das ilhas do estado do Rio de Janeiro. In: Branco JO, editor. Aves marinhas e insulares brasileiras: biologia e conservação. Itajaí: Editora da Univali; 2004. p. 83-100.

3. Efe MA. Aves marinhas das ilhas do Espírito Santo. In: Branco JO, editor. Aves marinhas e insulares brasileiras: bioecologia e conservação. Itajaí, SC: Editora da Univali; 2004. p. 101-18.

4. Efe MA, Musso C, Glock L. Sucesso reprodutivo de *Thalasseus sandvicensis eurygnathus* no Brasil. Biociencias. 2005;13:63-8.

5. Efe MA, Bonatto SL. Evaluation of the status of conservation of the Cabot's Tern (*Thalasseus acuflavidus*) in Brazil. Revista Brasileira de Ornitologia. 2011;19:358-63.
