# Supplementary material for: Environmental Predictors of Seabird Wrecks in a Tropical Coastal Area
Source: PLoS One. 2016 Dec 16;11(12):e0168717. doi: 10.1371/journal.pone.0168717 (PMC5161483; doi:10.1371/journal.pone.0168717)
Supplement: S3 Table — (DOCX) [file pone.0168717.s007.docx]

**S3 Table. Variance Inflation Factors for the predictor variables.**

| **Predictive variables** | **VIF 1** | **VIF 2** |
| --- | --- | --- |
| Oil spill | 1.11 | 1.10 |
| Onshore wind frequency | 1.98 | 1.48 |
| Distance surveyed | 1.34 | 1.14 |
| Chlorophyll-a concentration | 125.42 | 1.59 |
| V current | 1.78 | 1.57 |
| River outflow | 1.37 | 1.36 |
| Distance from breeding islands | 2.07 | 2.05 |
| Wind direction | 2.31 | 2.30 |
| Wind speed | 2.39 | 2.36 |
| Wave period | 2.49 | 2.39 |
| U current | 2.49 | 2.48 |
| Upwelling Index | 2.59 | 2.53 |
| Latitude | 2.86 | 2.58 |
| Wave height | 3.43 | 2.62 |
| Sea surface temperature | 3.15 | 2.93 |
| Water depth | 4.09 | - |
| k490 | 121.50 | - |

VIF 1 = Variance Inflation Factors with problematic values higher than 3. VIF 2 = Variance Inflaction factors after removing the variables water depth and the Diffuse Attenuation Coefficient at 490 nm wavelength (k490) of the data set.
