# Supplementary material for: Environmental Predictors of Seabird Wrecks in a Tropical Coastal Area
Source: PLoS One. 2016 Dec 16;11(12):e0168717. doi: 10.1371/journal.pone.0168717 (PMC5161483; doi:10.1371/journal.pone.0168717)
Supplement: S4 Table — Model goodness of fit to the data was evaluated using multiple indicators: chi-square test (p-values > 0.05 indicate relatively good model fits); Comparative Fit Index (CIF, values > 0.95 indicate good model fits); root mean square error (RMSE, values < 0.06 indicate good model fits) and the weighted root mean square residual (WRMR, values < 0.90 indicate good model fits). The fit indices indicate the Structural Equation Models for all the species met the standard criteria: CFI > 0.96, RMSE < 0.05, and WRMR < 0.65.] (DOCX) [file pone.0168717.s008.docx]

**S4 Table. The measures of goodness of fit indicate of the structural equation models for seabird stranding events in Brazil**. We evaluated model goodness of fit to our data using multiple indicators: chi-square test (p-values > 0.05 indicate relatively well model fits); Comparative Fit Index (CIF, values > 0.95 indicate good model fits); root mean square error (RMSE, values < 0.06 indicate good model fits) and the weighted root mean square residual (WRMR, values < 0.90 indicate good model fits). The fit indices indicate the structural equation models for all the species met the standard criteria: CFI > 0.96, RMSE < 0.05, and WRMR < 0.65.

| **Species** | **Chi-square** | **CFI** | **RMSE** | **WRMR** |
| --- | --- | --- | --- | --- |
| Brown booby | 15.93 | 0.96 | 0.05 | 0.65 |
| Magnificent frigatebird | 1.19 | 1.00 | 0.00 | 0.25 |
| Kelp gull | 11.27 | 0.99 | 0.03 | 0.62 |
| Cabot's tern | 1.69 | 1.00 | 0.00 | 0.25 |
